# Bovine Cartilage-Derived Type II Collagen Composite Scaffolds: Collagen Characterization, Physicochemical Properties, and In Vitro Chondrocyte Responses

**DOI:** 10.3390/jfb17030116

**Published:** 2026-02-28

**Authors:** Zihan Zhu, Ming Ju, Min Li, Wangang Zhang

**Affiliations:** 1 State key Laboratory of Meat Quality Control and Cultured Meat Development, College of Food Science and Technology, Nanjing Agricultural University, Nanjing 210095, China; 2022208033@stu.njau.edu.cn (Z.Z.); 2022208032@stu.njau.edu.cn (M.J.); 2 Delisi Group Co., Ltd., Weifang 262200, China; 13562680705@163.com

**Keywords:** type II collagen, bovine cartilage, composite scaffold, porous structure, cartilage tissue engineering, primary human chondrocytes

## Abstract

Type II collagen (CII), the major structural protein in the cartilage extracellular matrix, is a promising biomaterial for scaffold design in cartilage tissue engineering. In this study, high-purity CII was successfully extracted from bovine cartilage, an abundant by-product of cattle slaughter, and its amino acid composition, triple-helical conformation, and thermal stability were verified. CII was subsequently combined with silk fibroin (SF) and chitosan (CS) to fabricate three-dimensional (3D) porous scaffolds via freeze-drying. The pore structure, porosity, swelling behavior, mechanical properties and in vitro degradation characteristics were systematically evaluated. Scaffolds with favorable structural integrity, mechanical performance, and degradation rates were further evaluated biologically using human primary chondrocytes. All CII-based composite scaffolds supported chondrocyte growth and promoted early extracellular matrix deposition. Notably, the scaffold with a CII:SF:CS ratio of 7:3:1 showed the highest GAG/DNA content, accompanied by upregulated gene expression related to the cartilage phenotype (COL2A1, ACAN, and SOX9) and reduced expression of the dedifferentiation marker COL1A1, indicating improved phenotype maintenance. Overall, within the tested range, CII70 (CII:SF:CS = 7:3:1) represents a practical compromise between scaffold stability and in vitro chondrocyte-related outcomes, providing a basis for selecting CII/SF/CS formulations for cartilage tissue engineering.

## 1. Introduction

Articular cartilage repair remains a challenging clinical issue, often resulting from trauma and abnormal mechanical stress during activity and aging [1,2]. Cartilage exhibits poor self-repair ability after injury due to the lack of vascularization and innervation, along with the limited proliferative capacity of chondrocytes [3,4,5]. As of 2020, approximately 600 million people worldwide are affected by osteoarthritis, and its incidence is projected to increase by 60–100% until 2050 [6]. Conventional clinical strategies for cartilage repair, including conservative treatment and surgical treatment (e.g., bone stimulation drilling, autologous transplantation), may alleviate pain and restore joint function but rarely regenerate tissue structures [7,8,9]. Therefore, there is a growing demand for alternative strategies that can support cartilage regeneration and functional restoration.

Cartilage tissue engineering has been regarded as a promising strategy [10,11], typically integrating cells, growth factors and suitable scaffold materials [8]. Among available biomaterials, type II collagen (CII), which resembles the native extracellular matrix and helps maintain chondrocyte function, is especially appealing for scaffold construction in cartilage tissue engineering [12]. As the predominant collagen in cartilage, CII accounts for over 90% of total collagen and approximately 60% of the cartilage dry weight [3,13]. Bovine cartilage is an abundant and low-cost slaughter by-product, and a promising source of type II collagen for scaffold fabrication [14,15]. Additionally, CII cross-links with proteoglycans and other proteins, forming a stable network that helps maintain the integrity of the cartilage tissue, as well as support the chondrocyte adhesion, proliferation and functional maintenance [16,17].

Type II collagen is attractive for cartilage tissue engineering because it supports chondrocyte-matrix interactions and helps preserve cartilage-specific features [15]. Recent studies have also explored advanced fabrication routes, such as injectable and 3D-printable CII-containing hydrogels, to better control architecture and promote cartilage-like tissue formation [18]. Nevertheless, CII-based constructs often suffer from insufficient mechanical robustness and relatively fast degradation, motivating composite designs to improve overall performance [10,19,20,21,22]. Among these materials, chitosan and silk fibroin have been commonly incorporated into collagen-based systems. Chitosan exhibits good biocompatibility and a chemical structure similar to glycosaminoglycans (GAGs), and silk fibroin is commonly used to enhance the mechanical properties of scaffolds [10,22].

In this study, CII was extracted from bovine cartilage and systematically characterized in terms of its molecular composition, structural features, and thermal stability. CII was then blended with silk fibroin and chitosan to fabricate 3D porous composite scaffolds. This composite design was intended to combine the bioactivity of CII with the mechanical reinforcement and structural stability provided by SF and CS. However, systematic evidence remains limited on how the CII-to-SF ratio shapes porous architecture and physicochemical performance and how these features relate to primary chondrocyte outcomes. For example, Gao et al. [10] focused on a single CII-to-SF ratio (3:7) and emphasized scaffold degradation behavior in vitro and in vivo, without a ratio-series comparison. In contrast, Lin et al. [20] investigated multiple SF/CII ratios but in a membrane format rather than porous scaffolds. Accordingly, in the current study, a series of CII/SF/CS scaffolds with varying CII-to-SF ratios were prepared and evaluated in terms of pore architecture, mechanical properties, swelling behavior, and degradation profiles. The selected scaffolds were further assessed using primary human chondrocytes for proliferation, phenotype maintenance, and extracellular matrix synthesis. Overall, this work demonstrates a value-added conversion of bovine cartilage, an abundant slaughter by-product, into purified type II collagen and subsequently into functional composite scaffolds for cartilage tissue engineering.

## 2. Materials and Methods

### 2.1. Extraction of Type II Collagen from Bovine Cartilage

Fresh bovine cartilage was purchased from a local agricultural market in Nanjing, China. After the removal of residual muscle and connective tissue, the cartilage was dried at 40 °C for 24 h, and ground into powder. The powder was sieved to 40 mesh prior to further treatment.

Non-collagenous proteins were removed by suspending the cartilage powder in 0.1 M NaOH (5%, *w*/*v*) at 4 °C for 48 h. Defatting was carried out by treating the powder with 10% (*v*/*v*) n-hexane (1:5, *w*/*v*) at 4 °C for 24 h. Demineralization was subsequently performed by dispersing the powder in 0.25 M EDTA (1:10, *w*/*v*) at 4 °C for 4 d. After demineralization, the residue was washed and centrifuged (12,000× *g*, 4 °C). The collected collagen fibrils were freeze-dried and stored at −20 °C for further utilization.

CII was extracted by a modified method of Dai et al. [23]. The dried collagen fibrils were suspended in 0.5 M acetic acid containing pepsin (1 g/L) at 4 °C for 48 h. The supernatant was collected after centrifugation (12,000× *g*, 4 °C) and immediately adjusted to pH 7.5 with 6 M NaOH to inactivate the enzyme. Collagen was precipitated by gradual addition of sodium chloride to a final concentration of 3.0 M under continuous stirring and left to stand overnight at 4 °C. The precipitate was dissolved in 0.5 M acetic acid, which was gradually added until all the precipitate was dissolved, and dialyzed using tubing with a molecular weight cutoff of 8–14 kDa. The dialyzed solution was freeze-dried to obtain bovine cartilage-derived type II collagen and stored at −20 °C for further utilization.

### 2.2. Type II Collagen Characterization

#### 2.2.1. SDS-PAGE Analysis

The molecular weight and the purity of the extracted collagen were examined by SDS-PAGE. Collagen samples were prepared in loading buffer and separated on a discontinuous polyacrylamide gel composed of a 3% stacking gel and an 8% resolving gel using a vertical protein electrophoresis apparatus (Bio-Rad Laboratories, Hercules, CA, USA). After electrophoretic separation, the gels were stained with Coomassie Brilliant Blue, and the resulting protein bands were recorded using a gel documentation system.

#### 2.2.2. Amino Acid Composition

Approximately 10 mg of sample was hydrolyzed with 5 mL of 6 mol/L HCl in sealed tubes at 110 °C for 24 h. The residue was dissolved in 0.1 mol/L HCl, followed by filtration through a 0.22 μm membrane. An aliquot of the filtrate was analyzed using an automatic amino acid analyzer (Hitachi High-Tech Corporation, Tokyo, Japan) to determine the total amino acid composition. The determination was based on the 18 common amino acids detected by acid hydrolysis (hydroxyproline and hydroxylysine were not quantified in this analysis). The data were expressed as residues per 1000 residues based on the amino acids detected under the current analytical condition.

#### 2.2.3. Fourier Transform Infrared (FTIR) Analysis

The molecular structure of the collagen sponge was examined by FTIR (Nicolet iS10, Thermo Fisher Scientific, Madison, WI, USA). Spectra were collected with an average of 16 scans over a wavenumber range of 350–7800 cm^−1^ at a resolution of 0.09 cm^−1^. The resulting spectra were processed and plotted using Origin 8.6 software.

#### 2.2.4. Circular Dichroism (CD) Analysis

Collagen samples were dissolved in 0.5 mol/L acetic acid to a final concentration of 0.15 mg/mL. CD spectra were recorded using a CD spectrometer (Bio-Logic, Seyssinet-Pariset, France) under a nitrogen atmosphere, with 0.5 mol/L acetic acid being used as the baseline. Quartz cuvettes were thoroughly cleaned prior to measurement. The recorded spectra were smoothed and used for subsequent analysis.

#### 2.2.5. Differential Scanning Calorimetry (DSC) Analysis

The denaturation temperature of collagen sample was measured referred to the method of Wang et al. [24] using DSC (DSC 8000, PerkinElmer, Waltham, MA, USA). Collagen sample was dissolved in 0.5 mol/L acetic acid to obtain a final concentration of 1 mg/mL. Ultrapure water was used as the reference, and DSC measurements were performed from 25 to 85 °C at a heating rate of 1 °C/min to obtain the thermograms.

### 2.3. Scaffold Fabrication

Type II collagen extracted from bovine cartilage was dissolved in 0.01 M HCl. Chitosan and water-soluble silk fibroin were separately dissolved in deionized water. Composite scaffolds were prepared by mixing type II collagen, silk fibroin, and chitosan solutions at different volume ratios. Five formulations were designated as CII100, CII70, CII50, CII30 and CII0, corresponding to CII/SF/CS ratios of 10:0:1, 7:3:1, 5:5:1, 3:7:1, and 0:10:1, respectively. Final concentrations were 1% (*w*/*v*) for total protein and 0.1% (*w*/*v*) for chitosan. The mixtures were stirred at 4 °C until homogeneity and were transferred into 48-well plates, frozen at −80 °C for 24 h, and lyophilized for 48 h to obtain cylindrical porous scaffolds.

The scaffolds were crosslinked using a 1-ethyl-3-(3-dimethylaminopropyl) carbodiimide/N-hydroxysuccinimide system. Briefly, 2 mL of crosslinking solutions (EDC 50 mmol/L, NHS 25 mmol/L, 75% ethanol, *v*/*v*) were added to each well and allowed to react at 4 °C overnight. The scaffolds were then subjected to a second freeze-drying cycle (Martin Christ, Osterode am Harz, Germany). The dried scaffolds were stored at 4 °C until further use.

### 2.4. Physicochemical Properties of Scaffolds

#### 2.4.1. Scanning Electron Microscopy (SEM) Observation and Pore Size Analysis

Scaffold samples were sectioned, gold-sputtered, and observed by a field emission scanning electron microscope (Ultra-Plus, Zeiss, Oberkochen, Germany) to examine their internal morphology. Pore size was analyzed using ImageJ software (version 1.54g, National Institutes of Health, Bethesda, MD, USA) based on SEM images taken from the central regions of the scaffolds. For each group, at least 20 pores were randomly selected, and their maximum and minimum diameters were measured to calculate the average pore size.

#### 2.4.2. Porosity

Scaffold porosity was evaluated using a modified liquid displacement method with anhydrous ethanol. A known volume of ethanol (V_1_) was placed in a graduated cylinder, and the dried scaffold (*n* = 5 per group) was immersed for 5 min under vacuum until air bubbles were eliminated. The total volume was recorded as V_2_. After removing the scaffold, the remaining ethanol volume was recorded as V_3_. Porosity was calculated as P (%) = (V_1_ − V_3_)/(V_2_ − V_3_) × 100.

#### 2.4.3. Swelling Ratio

The swelling ratio of the scaffolds was evaluated by a gravimetric method. Freeze-dried scaffolds (*n* = 5 per group) were weighed to obtain the initial mass (m_1_) and then immersed in PBS (pH 7.4) for 24 h. After the gentle removal of surface water, the swollen mass (m_2_) was recorded. The swelling ratio (%) was calculated as (m_2_ − m_1_)/m_1_ × 100.

#### 2.4.4. Mechanical Properties

The compressive mechanical properties of the scaffolds were evaluated using a universal testing machine (Suns Technology Co., Ltd., Shenzhen, China). Scaffolds were cut into uniform cubic specimens (1 × 1 × 1 cm^3^). Five independent replicates (*n* = 5) were tested for each scaffold group. Prior to testing, samples were fully hydrated in phosphate-buffered saline (PBS, pH 7.4) at 37 °C until equilibrium was reached, and excess surface liquid was gently removed. Compression tests were performed in the hydrated state at a constant crosshead speed of 2 mm/min. Stress–strain curves were recorded up to 60% strain. The compressive stress (σ) was calculated using the formula: σ = F/A_0_, where F is the compressive load applied to the specimen and A_0_ is the initial cross-sectional area (1 cm^2^). Since the highly porous scaffolds were continuously compressed without exhibiting a distinct fracture point within the tested range, the compressive stress at 40% strain (σ40%) was specifically recorded as a representative value of the scaffold’s mechanical resistance. Young’s modulus was determined from the slope of the initial linear region (0–10% strain).

#### 2.4.5. In Vitro Degradation

The enzymatic degradation of the scaffolds was evaluated using an accelerated collagenase digestion assay. Freeze-dried scaffolds from each group were cut into comparable pieces (*n* = 5 per group), placed in 5 mL microcentrifuge tubes, and weighed to obtain the initial dry mass (m_1_). Each sample was then incubated in phosphate-buffered saline (PBS, pH 7.4) containing type II collagenase (Macklin, Shanghai, China; 0.5 mg/mL) at 37 °C under gentle shaking (100 rpm) for 60 h. After incubation, samples were collected by centrifugation, washed thoroughly with deionized water to remove residual enzyme, and vacuum-dried (or lyophilized) to constant weight. The final dry mass after digestion was recorded as m_2_. The degradation rate (%) was calculated as (m_1_ − m_2_)/m_1_ × 100.

### 2.5. Biological Performance of Scaffolds

#### 2.5.1. Cell Proliferation

Primary human chondrocytes (AW-CNH489) and a chondrogenic maintenance medium supplemented with proline, dexamethasone, and human TGF-β3 were purchased from Abiowell Biotechnology Co., Ltd. (Wuhan, China). The scaffolds were washed with 70% (*v*/*v*) ethanol (four times for 30 min each at 20 °C), followed by rinsing with sterile 0.9% saline (five times for 15 min each at 20 °C).

Chondrocytes were seeded onto the scaffolds in 48-well plates at a density of 1 × 10^5^ cells per well, with a final medium volume of 300 μL. The medium was refreshed every 3 d. Cell proliferation was assessed over a 14 d period. At 1, 4, 7, 10, and 13 d, the medium was removed and replaced with 300 μL of CCK-8 working solution (10%, *v*/*v*). After incubation at 37 °C for 4 h, 100 μL of the reaction solutions were transferred to a 96-well plate, and the absorbance was measured at 450 nm. Chondrocytes cultured without scaffolds served as the control. Three independent cell-seeded scaffolds were used per group at each time point (*n* = 3).

#### 2.5.2. GAG Content

The GAG content in cell-seeded matrices was assessed by commercial GAG ELISA kits (MREDA, Wuhan, China). The scaffolds sample were collected on the 7th and 14th d of the cultivation process (*n* = 3 per group). After incubation in papain digestion buffer at 60 °C for 16 h, the digested samples were centrifuged at 12,100× *g* for 15 min, and the supernatants were used for GAG determination. GAG content was normalized to the DNA content of each sample to account for differences in cell number among different scaffolds. DNA was quantified using a Hoechst 33258 fluorescence assay.

#### 2.5.3. Chondrogenic Gene Expression Analysis

Total RNA from the cell-seeded matrices was extracted using Trizol (Ambion, Thermo Fisher Scientific, Austin, TX, USA). Samples were collected after 7 d of culture. RNA concentration and purity were determined using a microvolume spectrophotometer (Thermo Fisher Scientific, USA). Equal amounts of RNA from each sample were reverse transcribed into complementary DNA (cDNA) using the HiScript III RT SuperMix kit (Vazyme, Nanjing, China). The reverse transcription reactions were performed in a gradient PCR thermal cycler. Quantitative real-time PCR was carried out using SupRealQ Purple Universal SYBR qPCR Master Mix (Vazyme, Nanjing, China) on a QuantStudio 6 Flex real-time PCR system. Glyceraldehyde-3-phosphate dehydrogenase (GAPDH) was used as the internal reference gene, and relative gene expression levels were calculated using the 2^−ΔΔCt^ method. Sequences used in this study are listed in Table 1. Gene expression analysis was performed using three independent biological replicates per group (*n* = 3).

### 2.6. Statistical Analysis

Unless otherwise specified, all experiments were performed with at least three independent replicates. Data are presented as mean ± SD. Statistical analyses were performed using SPSS (version 18.0, SPSS Inc., Chicago, IL, USA). Differences among groups were evaluated by one-way analysis of variance (ANOVA) followed by Tukey’s post hoc test for multiple comparisons. Statistical significance was set at *p* < 0.05. For letter-based annotations, bars that do not share a common letter are significantly different.

## 3. Results and Discussion

### 3.1. Isolation and Characterization of Bovine Type II Collagen (CII)

**Figure 1:**
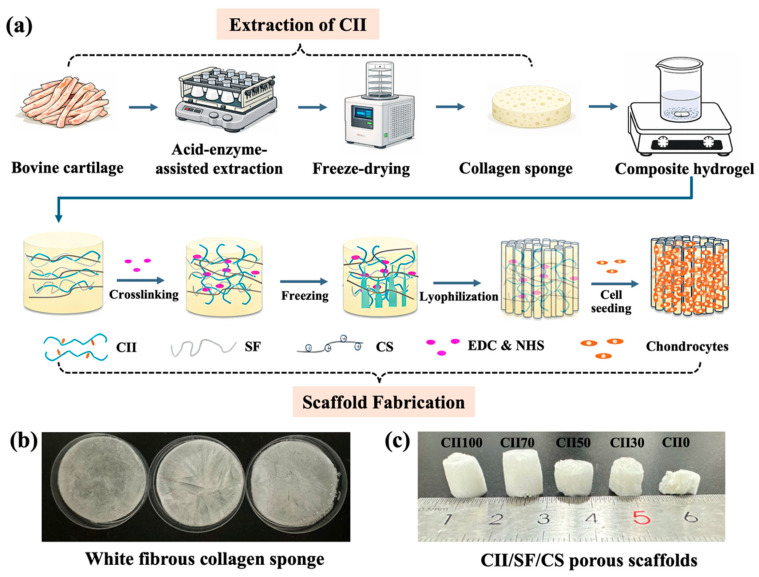
Schematic illustrations of scaffold fabrication: (**a**) schematic workflow illustrating the extraction of bovine cartilage-derived CII and the fabrication of CII/SF/CS scaffolds; (**b**) images of white fibrous collagen sponge derived from bovine cartilage; and (**c**) images of the CII/SF/CS porous scaffolds with different formulations. Arrows indicate the sequence of the experimental workflow.

There are many methods for collagen extraction, include acid extraction, enzyme extraction, and alkaline extraction. Enzyme extraction is generally preferred as it can improve collagen purity by removing non-helical telopeptide regions while maintaining a reasonable yield [25,26,27]. In this study, an acid-enzyme-assisted extraction approach was employed to isolate CII from bovine cartilage. As shown in Figure 1b, the extracted CII formed white fibrous sponges after freeze-drying. The characteristics of collagen extracted from bovine cartilage were systematically analyzed by amino acid composition analysis, SDS-PAGE, differential scanning calorimetry (DSC), Fourier transform infrared spectroscopy (FTIR), and circular dichroism (CD).

**Figure 2:**
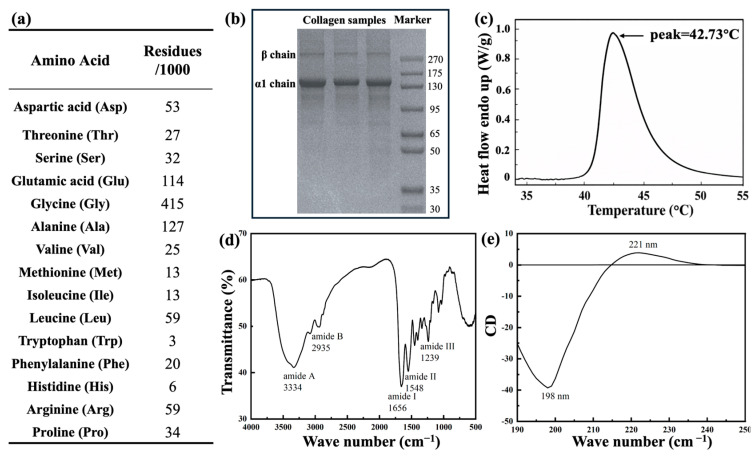
Characterization of bovine type II collagen (CII): (**a**) amino acid composition of CII; (**b**) SDS-PAGE pattern of the isolated CII, lanes 1–3 represent three independent replicates; (**c**) DSC thermogram; (**d**) FTIR analysis; and (**e**) CD analysis. The arrow indicates the denaturation peak temperature (Tmax).

A total of 16 amino acids were detected, with glycine being the most abundant residue, accounting for 359 residues per 1000 residues (Figure 2a). Glycine is periodically distributed within the collagen molecule and plays a significant role in the tight stacking of the triple helix and the stability of its spatial conformation [15,28]. Proline was also present at a relatively high level (124 residues/1000), which is known for maintaining the rigidity and conformational stability of the triple helix [23], followed by alanine (117 residues/1000) and glutamic acid (104 residues/1000). The amino acid composition observed in this study is consistent with previous reports on type II collagen derived from squid, chicken, and bovine tracheal cartilage [23,29,30].

SDS-PAGE analysis (Figure 2b) further confirmed the molecular characteristics of the isolated collagen. A dominant band was observed at approximately 130 kDa, corresponding to the α_1_(II) chain. Meanwhile, a weaker band with higher molecular weights was detected attributing to β-chain dimers. This band pattern is consistent with previously studies [17,29,31]. Notably, no obvious impurity bands were observed, indicating that the extracted CII possessed high purity.

As shown in the DSC thermogram (Figure 2c), the denaturation temperature of bovine cartilage-derived CII extracted in acetic acid solution was approximately 43.68 °C, which is comparable to the denaturation temperatures reported for bovine collagens (40–42 °C) in the literature [32]. Bovine collagen generally exhibits a higher denaturation temperature compared with marine collagens [33], reflecting superior thermal stability. This property allows bovine collagen to maintain its structural integrity under physiological conditions, thereby enhancing its processability for biomedical applications.

The structural integrity of bovine cartilage-derived CII was evaluated by FTIR (Figure 2d) and CD analyses (Figure 2e). The FTIR spectrum exhibited characteristic absorption peaks at 3334 cm^−1^ (amide A), 2935 cm^−1^ (amide B), 1656 cm^−1^ (amide I), 1548 cm^−1^ (amide II), and 1239 cm^−1^ (amide III). The amide I band is mainly related to C=O stretching vibrations of peptide bonds and is highly sensitive to changes in the secondary structure of collagen. The characteristic absorption bands in the range of 1200–1400 cm^−1^ are attributed to N-H stretching vibrations and the symmetric stretching of -COO- groups, which is a unique infrared spectral for collagen. This is related to the high content of glycine and characteristic amino acids (hydroxyproline and proline) in collagen, as well as the formation of a unique sequence of (Gly-Pro-Hyp)_n_. This spectroscopy pattern is similar to the previous study [29], suggesting that the triple-helical structure of collagen was largely preserved during extraction.

Circular dichroism (CD) spectroscopy further supported the retention of the triple-helical conformation. The spectrum showed the characteristic collagen profile, with a positive band around 221 nm and a negative band near 198 nm (Figure 2e), indicating that the extracted CII predominantly remained in an ordered helical state. In general, substantial disruption of the triple helix is associated with attenuation or loss of the positive band and a shift in the negative band toward longer wavelengths [34].

### 3.2. Fabrication of CII/SF/CS Composite Scaffolds

The overall workflow for the extraction of bovine cartilage-derived type II collagen and the fabrication of CII/SF/CS composite scaffolds is schematically illustrated in Figure 1a. The fabrication of scaffolds is a multistep process involving collagen extraction, purification, crosslinking, and scaffold construction. CII was blended with silk fibroin (SF) and chitosan (CS) to construct 3D porous scaffolds. SF was incorporated as a reinforcing protein phase to improve mechanical integrity of the scaffolds and mitigate excessive degradation, thereby providing sustained physical support during culture [10,20]. CS was introduced as a polysaccharide component to better resemble the glycosaminoglycan-rich cartilage matrix and improve scaffold formability. Moreover, its cationic property may facilitate intermolecular interactions with collagen networks and stabilize pore walls during swelling [22]. In this study, CS was kept constant, while the CII-to-SF ratio was varied to modulate pore architecture, mechanical performance, and degradation behavior, thereby identifying an optimized scaffold formulation with stable physicochemical performance and favorable chondrocyte responses.

Freeze-drying is a widely used technique for fabricating porous scaffolds from natural polymers, such as collagen, gelatin, and silk fibroin [15,35]. In this study, scaffolds with different compositions were fabricated by freeze-drying, followed by EDC/NHS crosslinking to enhance structural stability. As shown in Figure 1c, the CII/SF/CS scaffolds exhibited a typical 3D porous structure, confirming successful formation of an interconnected porous network through freeze-drying. Although native CII has been linked to potential autoimmune concerns, crosslinking has been reported to reduce its immunogenicity and alleviate adverse immune responses [36,37].

### 3.3. Physicochemical Properties of CII Scaffold

#### 3.3.1. Microstructure of Scaffolds

**Figure 3:**
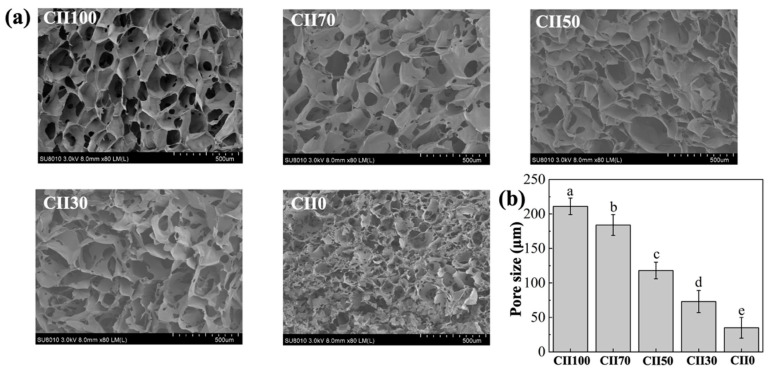
SEM images (**a**) and pore size (**b**) of CII/SF/CS scaffolds. The data in (**b**) are presented as mean ± SD (*n* = 5). Different letters indicate significant differences among groups (one-way ANOVA with Tukey’s post hoc test, *p* < 0.05).

All scaffold samples exhibited a porous morphology in Figure 3a and the average pore size was evaluated by Image J as shown in Figure 3b. This porous architecture provided a large specific surface area favorable for cell adhesion, migration, and proliferation. Scaffolds with higher CII content displayed more regular and well-defined pore structures, whereas those with lower CII content exhibited less uniform architecture, characterized by irregular pore shapes and partial pore closure.

Pore size is an important structural parameter that influences cell residence and tissue integration within porous scaffolds [38]. The CII100 scaffold (CII:SF:CS = 10:0:1) showed the largest pore size, with an average value of 211 ± 12 μm. As the proportion of CII decreased, a gradual reduction in pore size was observed, and the smallest pores were found in the CII0 scaffold (CII:SF:CS = 0:10:1), with an average size of 45 ± 15 μm. It has been reported that pore diameters larger than 100 μm were generally considered beneficial for cell adhesion, whereas for some biodegradable scaffold systems, smaller pore sizes can also effectively support cell growth [39,40]. Accordingly, the CII100, CII70, and CII50 scaffolds fall within pore size ranges were commonly considered to be permissive for cell attachment and cell residence in degradable scaffold systems. The hydroxyl groups of hydroxylysine in CII can interact with carbohydrate moieties forming a stable network through intermolecular crosslinking. This network not only resists the swelling pressure induced by hydration but also retains cartilage with favorable compressive resistance and viscoelastic properties [41].

#### 3.3.2. Scaffold Porosity

**Figure 4:**
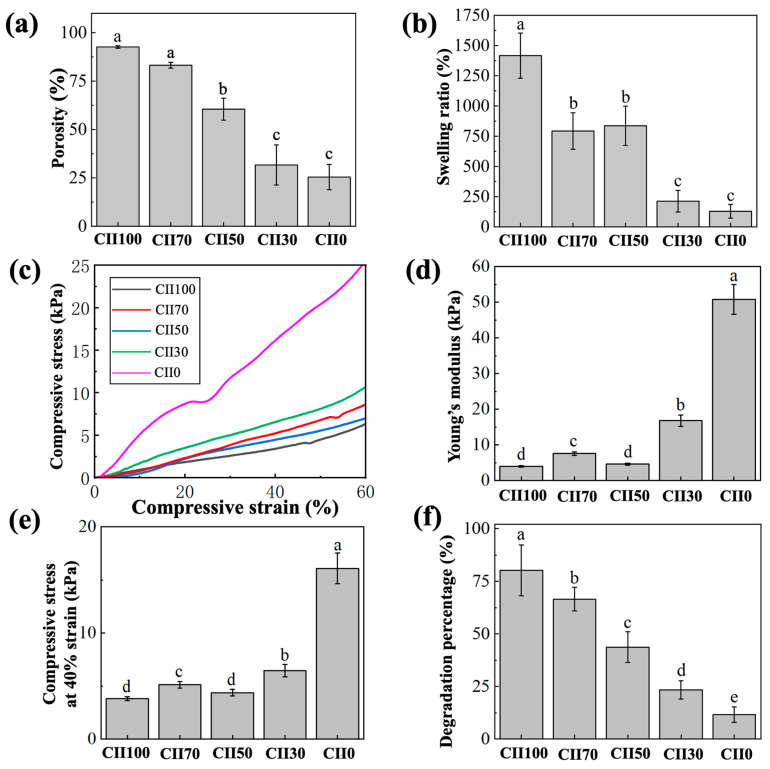
Physicochemical properties of CII/SF/CS scaffolds: (**a**) porosity; (**b**) swelling ratio; (**c**) compressive stress–strain curves measured under hydrated conditions; (**d**) Young’s modulus calculated from 0 to 10% strain; (**e**) compressive stress at 40% strain; and (**f**) degradation percentage. Data are presented as mean ± SD (*n* = 5). Different letters indicate significant differences among groups (one-way ANOVA with Tukey’s post hoc test, *p* < 0.05). Groups sharing at least one common letter are not significantly different (*p* > 0.05).

The porosity of the scaffolds is summarized in Figure 4a. The CII100 scaffold showed the highest porosity (92.63 ± 0.67%) and was not significantly different from CII70 (83.20 ± 1.21%; *p* > 0.05). High porosity facilitates the diffusion of nutrients into the scaffold interior and the removal of cellular metabolic waste. A porosity exceeding 80% is generally considered desirable [2]. In the present study, porosity showed a decreasing trend with decreasing CII content. CII50 was significantly lower than CII100 and CII70, whereas CII30 and CII0 exhibited the lowest porosity with no significant difference between them. Notably, there is also evidence suggesting that the optimal porosity may vary across different stages of tissue repair, and a single ideal pore structure has yet to be clearly defined [39]. In addition, the porosity of scaffolds constructed by freeze-drying is strongly influenced by the water content of the precursor gel. The pronounced hydrophilicity of CII may increase gel water retention before freezing. A higher water content may facilitate the formation and growth of ice crystals during the freezing step, and the subsequent sublimation of these ice crystals ultimately leads to the development of a more porous scaffold structure [12].

#### 3.3.3. Swelling Behavior

The swelling ratio evaluates the capacity of tissue engineering scaffolds to absorb water under culture medium or physiological conditions. As shown in Figure 4b, the CII100 scaffold exhibited the highest swelling ability, with a value of 1416.27 ± 186.60%. The water uptake capacity is closely associated with the retention of nutrients after implantation, whereas excessive swelling may induce structural deformation and decrease scaffold stability [42].

In this study, consistent with the findings of Lin et al. [20], swelling increased with increasing CII content. This behavior may be explained by the intrinsic hydrophilicity of type II collagen, containing abundant hydrophilic functional groups that facilitate water binding and retention. While higher CII content improves hydration, excessive swelling may compromise dimensional stability, suggesting a need to balance water absorption capacity with structural integrity in scaffold design.

#### 3.3.4. Mechanical Performance

Mechanical performance determines whether the scaffold can provide adequate structural support, and also influences cell adhesion, proliferation, and differentiation [2,42]. The compressive stress–strain curves are shown in Figure 4c. All groups exhibited an approximately linear increase in stress at low strains, followed by a steeper rise at higher strains. Because no distinct failure point was observed within the tested strain range (up to 60%), mechanical resistance was compared using the compressive stress at 40% strain (σ40%). Quantitatively, Young’s modulus (0–10% strain; Figure 4d) and σ40% (Figure 4e) were generally higher in the SF-rich scaffolds, with CII0 showing the highest values. This is consistent with the SEM observations (Figure 3a), where higher SF content was associated with a denser pore-wall framework, which likely strengthened the load-bearing backbone after crosslinking.

Notably, the intermediate compositions (e.g., CII70 and CII30) maintained moderate stiffness and compressive resistance while avoiding the pronounced stiffening observed in CII0. This balance is relevant for cartilage scaffolds, where excessive densification and high stiffness may reduce pore accessibility and be less favorable for uniform cell distribution and extracellular matrix deposition [43]. Therefore, although SF-rich scaffolds improved mechanical retention, compositions with intermediate SF content offer a more practical compromise between mechanical support and a permissive porous microenvironment for chondrocyte-related applications.

#### 3.3.5. Biodegradation Behavior

The biodegradation behavior of scaffolds directly affects their performance during tissue regeneration. The in vitro degradation of the CII/SF/CS scaffolds was evaluated in collagenase solution at 37 °C for 60 h (Figure 4f). This accelerated enzymatic challenge was used to compare formulation-dependent stability. The CII100 scaffold exhibited the highest degradation percentage 80.2 ± 12.1%, followed by the CII70 scaffold (66.5 ± 5.6%) and the CII50 scaffold (43.7 ± 7.3%). In contrast, the CII30 and CII0 scaffolds showed markedly lower degradation percentages with 23.4 ± 4.4% and 11.5 ± 3.7%, respectively. Significant differences were observed among all groups (*p* < 0.05). This trend is consistent with previous reports showing that CII was relatively susceptible to degradation, while SF could effectively retard scaffold degradation [20]. Excessively rapid degradation may lead to an early loss of mechanical support, whereas slow degradation may hinder tissue ingrowth and remodeling [20]. The observed degradation behavior is likely shaped by multiple factors that co-vary across the formulations. For instance, higher CII content may increase water affinity and, together with a more open porous structure (as suggested by SEM/porosity results), facilitate solution penetration and enzyme access. Notably, CII70 showed an intermediate mass loss under collagenase challenge, suggesting a moderate level of enzymatic stability among the tested formulations. Similar observations have been reported in SF-based composite systems, where the incorporation of collagen components enabled degradation modulation while improving mechanical performance [10]. A controlled degradation manner that the degradation process matches new tissue regeneration is desirable in tissue engineering [44].

### 3.4. Biological Properties of CII Scaffolds

Primary chondrocytes and mesenchymal stem cells (MSCs) are seed cells that are typically used in cartilage tissue engineering. MSCs are widely used to evaluate the chondrogenic induction potential of scaffolds [15], whereas primary chondrocytes are commonly used to assess phenotype maintenance of mature cartilage cells. In this study, primary human chondrocytes were selected as the seed cells and the culture duration was limited to 14 d for the in vitro evaluation. The culture period was selected based on a previous study showing that human primary chondrocytes tended to undergo dedifferentiation during prolonged in vitro culture (approximately 14–28 d), characterized by an increased expression of type I collagen and a gradual loss of cartilage-specific phenotypes [45].

#### 3.4.1. Proliferation of Chondrocytes

**Figure 5:**
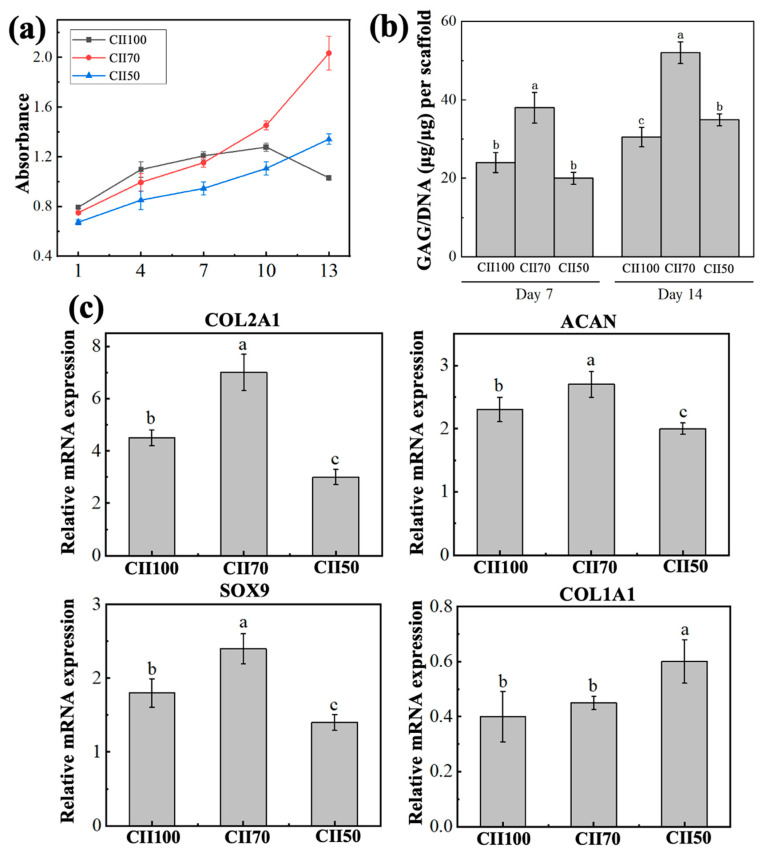
Biological properties of CII scaffolds: (**a**) cell proliferation of scaffolds; (**b**) GAG content; and (**c**) relative mRNA expression of COL2A1, ACAN, SOX9 and COL1A1. Data are presented as mean ± SD (*n* = 3). Different letters indicate significant differences among groups (one-way ANOVA with Tukey’s post hoc test, *p* < 0.05).

Primary human chondrocytes were seeded onto different CII/SF/CS scaffolds and cultured for 14 d, and cell proliferation was evaluated using the CCK-8 assay (Figure 5a). Chondrocyte proliferation on 3D scaffolds showed distinct patterns depending on scaffold composition. Although the CII100 scaffold contained the highest proportion of CII and exhibited a relatively rapid increase in cell proliferation at the early culture stage, its proliferation rate declined after 7 d, which coincided with higher mass loss indicating reduced scaffold integrity and physical support for continued cell expansion. This result suggests that a higher content of CII does not necessarily translate into improved cell proliferation.

The CII70 (CII:SF:CS = 7:3:1) scaffold showed a continuous increase in CCK-8 absorbance throughout the entire culture period. Notably, chondrocytes cultured on the CII70 scaffold maintained a clear upward growth trend after 10 d. By d 10 and 13, the cell proliferation level of the CII70 group was significantly higher than those of both the CII100 and CII50 groups (*p* < 0.05). This observation may reflect a combination of its pore architecture, porosity and composition-related factors. An appropriate 3D porous structure within scaffolds may facilitate cell adhesion and sustained proliferation [46].

The CII50 scaffold (CII:SF:CS = 5:5:1) also supported sustained chondrocyte proliferation, whereas the overall proliferation level remained lower than that was observed for the CII70 scaffold. Collagen-based matrices can present integrin-binding motifs (e.g., GFOGER-like sequences), which may contribute to cell adhesion and spreading via integrin-mediated pathways. Adequate early cell adhesion and spreading are generally regarded as important prerequisites for cell survival and sustained proliferation [22].

#### 3.4.2. Extracellular Matrix Production and Chondrogenic Gene Expression

Figure 5b shows the GAG/DNA values of the three CII/SF/CS scaffolds, with significant differences being observed among groups at both 7 and 14 d. The GAG/DNA content of the CII70 scaffold was significantly higher than that of the CII100 & CII50 scaffolds (*p* < 0.05), whereas no significant difference was observed between the CII100 & CII50 scaffolds at 7 d. After 14 d of culture, the GAG/DNA contents increased in all scaffold groups compared with 7 d. Among them, the CII70 scaffold continued to exhibit the highest level of GAG deposition, which was significantly greater than those of the CII100 & CII50 scaffolds (*p* < 0.05).

Figure 5c presents the mRNA expression levels of cartilage phenotype- and matrix synthesis-related genes in chondrocytes cultured on different CII/SF/CS scaffolds for 7 d. The expression levels of COL2A1, ACAN, and SOX9 were highest in the CII70 scaffold, while CII100 was intermediate and CII50 was lowest (*p* < 0.05). In contrast, the expression pattern of the dedifferentiation-associated gene COL1A1 differed from that of the cartilage-specific genes, with the CII50 scaffold showing a significantly higher COL1A1 expression than those of the CII100 & CII70 scaffolds (*p* < 0.05), while no significant difference was observed between the CII100 & CII70 scaffolds.

The GAG/DNA and qPCR results indicate that all CII-based scaffolds supported early matrix deposition and showed a trend toward maintaining cartilage-related gene expression. This observation is consistent with the results of previous studies [17,22,47]. Among the tested formulations, CII70 was associated with higher GAG/DNA and a more favorable pattern of cartilage-related gene expression, which may reflect a combination of pore architecture, porosity and cell–matrix interactions associated with the material composition. Zhang et al. [48] systematically investigated the influence of collagen sponge pore sizes (150–250, 250–350, 350–425, and 425–500 μm) on chondrocyte behavior and reported that the 150–250 μm group showed higher COL2A1 and ACAN expression and increased sGAG/DNA ratios. Similar trends have also been observed in non-collagen porous scaffold systems [49]. Pores in the 150–250 μm range may facilitate cell residence and local cell–cell interactions that contribute to the maintenance of the chondrocytic phenotype. In addition, variations in pore size may indirectly influence chondrocyte behavior by altering scaffold stiffness [5]. It should be noted that other structural factors, including pore interconnectivity, material composition, and their combined effects, also play important roles in regulating chondrocyte behavior. Therefore, the influence of pore size on chondrocyte phenotype maintenance and matrix synthesis should be interpreted with appropriate caution. Across the formulation series, higher CII content tended to increase porosity and swelling, whereas CII100 showed weaker hydrated mechanical retention and lower enzymatic stability. Within the tested range, CII70 showed the most workable balance between scaffold stability and chondrocyte-related performance. Although internal cell distribution was not directly assessed, the mean pore size (>100 μm) together with high porosity provided a pore network generally favorable for mass transport under 3D culture conditions. Consistently, chondrocytes showed sustained metabolic activity and increased GAG deposition over 14 d, suggesting that the scaffold environment can maintain cell viability and matrix synthesis in this 3D porous microenvironment. Nevertheless, without direct cross-sectional evidence, the uniformity of internal cell distribution warrants further evaluation in future studies.

## 4. Conclusions

In this study, high-purity type II collagen (CII) was extracted from bovine cartilage and used to fabricate a series of porous CII/SF/CS composite scaffolds by varying the CII-to-SF ratio. The scaffolds exhibited composition-dependent physicochemical properties, including pore architecture, porosity, swelling behavior, hydrated compressive performance, and collagenase-induced degradation stability.

Overall, CII70 (CII:SF:CS = 7:3:1) represents a practical compromise between scaffold physicochemical properties and in vitro chondrocyte performance. Under the tested conditions, CII70 supported higher chondrocyte proliferation and GAG/DNA deposition and showed a more favorable cartilage-related gene expression pattern than the other evaluated formulations, while maintaining moderate mechanical retention and enzymatic stability. Future work will focus on validating performance in vivo, along with direct assessment of the cell distribution within the scaffold and longer-term cartilage repair efficacy.

## Figures and Tables

**Table 1:** Primer sequence of target genes.

Gene	Forward Primer (5′–3′)	Reverse Primer (5′–3′)
COL2A1	AGGGCAACAGCAGGTTCAC	TGTCCACACCGAATTCCTG
COL1A1	GAGGGCCAAGACGAAGACAT	CAGATCACGTCATCGCACAAC
ACAN	TGGAGGTGCTGTTGACTTCC	GAGTAGCAGGAGGTGGGTGT
SOX9	AGCGAACGCACATCAAGAC	CTGTAGGCGATCTGTTGGGG
GAPDH	GAAGGTGAAGGTCGGAGTC	GAAGATGGTGATGGGATTTC

## Data Availability

The original contributions presented in the study are included in the article, further inquiries can be directed to the corresponding author.
